# Area-Level Associations between Built Environment Characteristics and Disability Prevalence in Australia: An Ecological Analysis

**DOI:** 10.3390/ijerph17217844

**Published:** 2020-10-26

**Authors:** Nicola Fortune, Ankur Singh, Hannah Badland, Roger J. Stancliffe, Gwynnyth Llewellyn

**Affiliations:** 1 Centre of Research Excellence in Disability and Health, University of Melbourne, Parkville, VIC 3010, Australia; ankur.singh@unimelb.edu.au (A.S.); hannah.badland@rmit.edu.au (H.B.); roger.stancliffe@sydney.edu.au (R.J.S.); gwynnyth.llewellyn@sydney.edu.au (G.L.); 2 Centre for Disability Research and Policy, University of Sydney, Lidcombe, NSW 2141, Australia; 3 Centre for Health Equity & Centre for Epidemiology & Biostatistics, Melbourne School of Population and Global Health, University of Melbourne, Carlton, VIC 3053, Australia; 4 Centre for Urban Research, RMIT University, Melbourne, VIC 3000, Australia

**Keywords:** disability, liveability, social determinants of health, accessibility, geographic variation

## Abstract

The importance of health-promoting neighborhoods has long been recognized, and characteristics of local built environments are among the social determinants of health. People with disability are more likely than other population groups to experience geographic mobility and cost restrictions, and to be reliant on ‘opportunity structures’ available locally. We conducted an ecological analysis to explore associations between area-level disability prevalence for people aged 15–64 years and area-level built environment characteristics in Australia’s 21 largest cities. Overall, disability was more prevalent in areas with lower walkability and lower local availability of various neighborhood amenities such as public transport, healthier food options, public open space, physical activity and recreation destinations and health and mental health services. These patterns of lower liveability in areas of higher disability prevalence were observed in major cities but not in regional cities. Our findings suggest that geographically targeted interventions to improve access to health-enhancing neighborhood infrastructure could reduce disability-related inequalities in the social determinants of health.

## 1. Introduction

The importance of health-promoting neighborhoods has long been recognized and is the focus of much policy attention at national and international levels [1,2,3]. Social determinants of health—the upstream factors that affect health through the conditions in which people are born, grow, live, work and age and that are, in turn, shaped by political, social and economic forces [4]—include characteristics of physical built environments to which people are exposed in their everyday lives. Such characteristics include, but are not limited to, levels of access to local employment, education, public open spaces, public and active transport infrastructure, affordable housing, and social infrastructure services [5,6,7,8].

Evidence showing associations between the built environment and health outcomes is mounting [9,10]. For example, empirical studies have shown local access to and diversity of social infrastructure is associated with subjective wellbeing [5]; neighborhood attributes such as local shops and the presence of footpaths and cycle paths are associated with levels of physical activity [11,12]; and local availability of healthy and fast-food outlets is associated with diet [13,14].

Compared with people without disability, people with disability experience greater disadvantage with respect to well-established social determinants of health; for example, they are more likely than those without disability to experience poverty [15,16,17,18,19], violence [20,21], social exclusion [15], housing insecurity [18,19], unemployment and economic inactivity [18,19,22]. As well as evidence of associations between disability and disadvantage at the individual level, there is evidence of a geographic dimension to disability-related disadvantage. Several studies have shown that people with disability tend to live in more socially disadvantaged areas than those without disability [23,24,25,26,27]. Such spatial patterning in exposure to social determinants of health, including those related to built environment characteristics, can potentially serve to exacerbate inequalities.

The concept of ‘liveability’ aligns with the social determinants of health, and has been used in the context of identifying, measuring and intervening to improve aspects of built environments that support health and wellbeing, particularly in urban contexts. A liveable neighborhood has been defined as ‘one that is safe, attractive, socially cohesive and inclusive, and environmentally sustainable; with affordable and diverse housing linked by convenient public transport, walking and cycling infrastructure to employment, education, public open space, local shops, health and community services, and leisure and cultural opportunities’ [28] (p.138). Sets of spatial indicators and composite indices have been developed to operationalize this definition and measure and monitor cities over time [8,9,28,29]. Liveability indicators can also be used to inform policy development and evaluate policy implementation. For example, in Australia a set of national liveability indicators has been developed covering the domains of walkability, public transport, public open space, housing affordability, employment, food environments, and alcohol environments in an effort to measure spatial inequities in liveability within and between cities [3]. Increasingly, new sources of spatial data and powerful software tools are making it possible to quantify aspects of the built environment at fine-grained spatial scales. When linked to other datasets, such spatial data can be used to explore associations with social, economic and health outcomes [30].

Liveability also has important links with the concept of accessibility in relation to people with disability. Accessibility is a vital principle in achieving equity for people with disability as articulated in Article 9 of the Convention on the Rights of Persons with Disabilities (CRPD) [31]. In its interactive, biopsychosocial model of disability, the World Health Organization’s International Classification of Functioning, Disability and Health (ICF) recognizes the crucial importance of environmental factors, which impact on all components of functioning and disability [32]. Characteristics of the physical, social and attitudinal environments in which people conduct their lives can facilitate functioning or pose barriers that serve to restrict people’s participation in various aspects of life. In Australia, ‘inclusive and accessible communities’ is the first of six areas of policy action identified in the National Disability Strategy 2010–2020 [33]. Characteristics of the built environment identified as key for achieving liveable neighborhoods are also necessary for ensuring physical accessibility for people with disability to enable participation in work, education, social and civic life [34].

There is a growing body of research concerning the effects of accessibility-related factors of built environments in relation to health, wellbeing and participation in different life areas for people with disability [35,36,37,38,39,40,41,42,43,44]. However, to date there has been relatively little research specifically investigating built environment exposures for people with disability at a population level taking a social determinants of a health perspective. Thus, there is limited evidence on the extent to which people with disability may reside in less ‘liveable’ neighborhoods and whether geographic variation in access to services and infrastructure serves to increase or decrease inequalities in the social determinants of health between people with and without disability. Such knowledge is critical to inform policies and interventions seeking to achieve inclusive societies in which people with disability can fulfil their potential as equal citizens [33].

In Australia, people living in regional and remote areas often have poorer health and welfare outcomes and higher rates of exposure to health risk factors than people living in major cities [45]. There is evidence that, for people with disability, access to health services and disability support services varies geographically, with higher levels of service access in major cities and lower levels in regional and remote areas [46,47,48]. It is not currently known whether any geographic associations between disability prevalence and local built environment characteristics differ between major cities and other areas in Australia.

Research taking an explicitly geographic approach and using area-level analyses can complement evidence from individual-level analyses about disability-related inequalities in exposure to social determinants of health and built environment impacts on the health and wellbeing of people with disability. Evidence of spatially patterned inequalities can potentially inform geographically targeted and tailored interventions to reduce inequalities. An ecological analysis approach, using geographic areas as the unit of analysis, is valuable for identifying spatial associations between exposures and outcomes defined at area-level. An ecological study design is one in which the units of analysis are groups, not individuals [49]. Ecological analyses do not aim to investigate causal processes or to shed light on associations at an individual level, but can be used to build an understanding of spatial patterning and heterogeneity of contextual social determinants of health at a population level [50,51].

In this paper, we investigate associations between disability prevalence among people aged 15 to 64 years and built environment characteristics for local areas within Australia’s 21 largest cities, which captures 80% of the Australian population aged 15–64 years. Our hypothesis is that people with disability experience geographically-mediated inequality in exposure to social determinants of health that are related to the built environment.

We address the following research questions:

1. Is area-level disability prevalence associated with area-level built environment characteristics such as walkability, healthy food environment, distance to social infrastructure services, proximity to public open space and availability of public transport?
2. Do associations between area-level disability prevalence and area-level built environment characteristics differ for major cities versus regional cities?

We discuss the policy implications of our findings in terms of the potential for targeted built environment interventions to reduce inequalities in the social determinants of health for people with disability in Australia.

## 2. Materials and Methods

### 2.1. Data Sources

To address our research questions, we used area-matched data from two sources: (i) the 2016 Australian Census of Population and Housing and (ii) spatial indicators of built environment characteristics for Australia’s 21 largest cities. The Australian Census is conducted by the Australian Bureau of Statistics (ABS) every five years and since 2011 it has included questions about disability, as described below. Census data are available for analysis at different levels of geographic aggregation. This makes it possible to conduct analyses exploring spatial associations between disability prevalence and other factors of interest for which sources of geocoded data are available, such as service locations [52,53]. The spatial indicators have been theoretically informed and empirically-tested with Australian adults [29].

### 2.2. Outcome

Our outcome was area-level prevalence of disability among people aged 15–64 years, sourced from the 2016 Australian Census of Population and Housing. The Census includes a ‘need for assistance with core activities’ module, comprising a set of four questions to identify people requiring assistance with self-care, mobility and/or communication due to a disability, long term health condition or the effects of old age [54]. We calculated disability prevalence as individuals with a core activity need for assistance as a percentage of all those who responded to this module; individuals who did not respond were excluded from the calculation of disability prevalence. Data were extracted using Census TableBuilder (Australian Bureau of Statistics, Canberra ACT, Australia) [55,56].

We chose to focus on disability prevalence in the population aged 15 to 64 years because this is the age range commonly referred to as ‘working age’. As well as the expectation that a high proportion of people with disability between the ages of 15 and 64 years will be in the labor force, many disability-specific services such as Disability Employment Services are targeted to people in this age group. Disability prevalence increases steeply in older age groups due to disability acquisition associated with ageing [57]. Compared with people of working age, it may be expected that both adults in older age groups and children aged under 15 have different types and patterns of interaction with their local environment, and important built environment features for health and wellbeing may differ.

### 2.3. Exposures

Exposures were spatially-derived built environment indicators for local areas within Australia’s 21 largest cities, covering 80% of the Australian population aged 15–64 years; the remaining 20% live in rural and remote areas for which these data were not available. Data for built environment characteristics were produced by the Healthy Liveable Cities Group in the Centre for Urban Research, RMIT University, using an Esri geographic information system (GIS) (ArcGIS Desktop 10.6, Version 10.6.0.8321, License: Advanced; Esri, Redlands CA, USA) and a Postgres database (PostgreSQL version 9.2.4, Server version 9.6; PostgreSQL Global Development Group). Each indicator was initially calculated for individual sample points (or ‘dwellings’, identified as unique address locations within residential zoned urban areas), then aggregated to provide summary measures at larger administrative geographic scales. Distances were calculated as the length of road network walkable by pedestrians from the sample point to the destination.

Data for this study were obtained for 27 built environment measures relating to food environment (six measures), social infrastructure services (13 measures), walkability (five measures), public open space (two measures) and public transport (one measure). From the initial list of 27 built environment characteristics, 18 were selected to examine associations with disability prevalence. Based on an evaluation of correlation coefficients and indicator definitions, we selected two food environment indicators and one indicator each for walkability and proximity to open space (see Tables S1–S3 in Supplementary File for correlation coefficients).

Appendix Table A1 describes how the 18 indicators selected for analysis were scored. Further detail on the calculation of the built environment indicators is available in the Australian Urban Observatory [58].

### 2.4. Covariates

**Figure 1:**
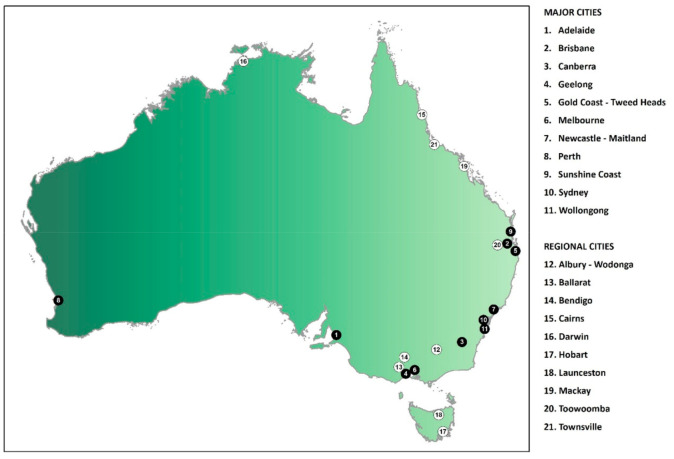
Map of Australia showing the location of the 11 major cities and 10 regional cities included in the analysis.

City type was considered as an effect modifier of the association between built environment characteristics and disability prevalence. The 21 cities for which the built environment indicator data were available were grouped into two categories, major cities (1314 Statistical Area Level 2s (SA2s)) and regional cities (172 SA2s) (Figure 1). This was done using the Remoteness Areas Structure within the Australian Statistical Geography Standard (ASGS), which divides Australia into five classes of remoteness based on a measure of relative access to services [59]. For each city, 2016 Census data were used to determine the proportion of the total population in each ASGS remoteness category. Cities for which the majority of the population lived in the ‘major cities’ ASGS category were designated ‘major cities’; cities for which the majority of the population lived in the ‘inner regional’ or ‘outer regional’ ASGS categories were designated ‘regional cities’.

### 2.5. Level of Geographic Aggregation

Statistical Area Level 2 (SA2) was the geographic level of aggregation used for examining associations between built environment characteristics and disability prevalence. This spatial unit is defined within the Main Structure of the ASGS, a hierarchical structure produced by the Australian Bureau of Statistics to enable the publication of statistics that are comparable and spatially integrated. With an average population of approximately 10,000 people, SA2s are designed to reflect functional areas that represent a community that interacts together socially and economically [60]. We selected SA2 as the geographic unit of analysis rather than the smaller SA1 unit (population between 200 and 800 people). Disability prevalence at the SA1 level was not sufficiently reliable due to the small number of people with disability in some SA1s. The larger SA3 geographic unit (population between 30,000 and 130,000 people) did not provide sufficient granularity to answer our research questions. We excluded 12 SA2s for which fewer than 500 people aged 15–64 years responded to the Census’ ‘need for assistance with core activities’ module.

### 2.6. Statistical Analysis

We conducted a descriptive analysis to report the central tendency and spread of built environment exposures and outcomes and analyzed associations between built environment characteristics and disability prevalence in three steps.

Step 1: For parsimony, we selected fewer built environment characteristics from the available list to qualify as independent exposures. To achieve this, within each group of indicators with two or more qualitatively similar indicators (i.e., food environment, walkability and proximity to public open space), we examined correlations to identify those likely to capture similar constructs. For pairs of indicators, the higher the correlation coefficient, the more likely it is that one indicator variable will predict the other. Therefore, where indicators showed high correlation, we selected the one identified as the most comprehensive and adequate based on the definition.

Step 2: After testing the assumption of normality for disability prevalence, separate linear regression models were fitted for each exposure selected in Step 1 and disability prevalence. Regression models were fitted with transformation of z-score standardized values of each exposure. Z-score standardization made it possible to compare the magnitude of association across exposures using a common scale.

Step 3: To examine effect modification by city type, for each exposure for which we found an association with disability prevalence in Step 2, an interaction term was fitted between exposure and city type using regression models. Where significant interaction terms were observed, we stratified the regression analyses to examine for variations in the association by city type.

All analyses were carried out in Stata v16 (Stata Statistical Software: Release 16. College Station, TX: StataCorp LLC).

### 2.7. Ethics

Ethical approval was not required for this study.

## 3. Results

Table 1 summarizes the descriptive statistics for the built environment variables.

Overall, we analyzed data for 1486 SA2s across Australia’s 21 largest cities, covering 80% of the Australian population aged 15–64 years; 88% of SA2s were in major cities, 12% were in regional cities. The mean prevalence of disability across SA2s was 3%, ranging from 0% to 12% (Table 1).

Associations between built environment characteristics and SA2 disability prevalence were found for multiple variables (see Table 2).

For indicators based on average distance measures (e.g., to social infrastructure services), positive associations meant greater average distances to destinations of interest (and thus lower levels of access) in SA2s with a higher disability prevalence; therefore, increased liveability with a higher disability prevalence was indicated by a negative (or inverse) association for distance variables. For indicators based on a number or per cent (e.g., number of healthier food options or percentage of dwellings within 400 meters of public open space), positive associations meant a greater number or percentage (and thus higher levels of access) in those SA2s with a higher disability prevalence.

Among the eight variables positively associated, the largest effect size was for distance to closest physical activity and recreation destination (i.e., greater distance in areas with a higher disability prevalence) and the smallest effect size was for distance to the closest pharmacy (Table 2). A unit increase in the *z*-score of distance to closest physical activity and recreation destination was associated with a mean change of 0.29 percentage points (95% CI: 0.22, 0.37) in disability prevalence. Similarly, a unit increase in the *z*-score of distance to the closest pharmacy was associated with a mean change of 0.10 percentage points (95% CI: 0.02, 0.17) in disability prevalence. Ranked in order of decreasing effect size, other variables that were positively associated were the average distance to the closest family counselling service, psychology service, adult mental health service, dentist, generalist counselling service, activity center, hospital and general practitioner. As all of these built environment characteristics were quantified using distance, a positive beta coefficient indicated greater distance and thus decreased liveability in SA2s with higher disability prevalence.

Five built environment characteristics were found to be inversely associated with the prevalence of disability, denoted by negative beta coefficients (Table 2). Ranked in order of decreasing effect sizes, these were public transport availability, the number of healthier food options, walkability index, public open space proximity and distance to a Disability Employment Service. Among these, a unit increase in the z-score of public transport availability was associated with a 0.36 percentage point lower mean prevalence of disability (95% CI: −0.43, −0.29) while a unit increase in the z-score of distance to a Disability Employment Service was associated with a 0.09 percentage point lower mean prevalence of disability (95% CI: −0.16, −0.01). The interpretation of these findings was complicated by the way these built environment characteristics were measured. Higher values for public transport availability, the number of healthier food options, walkability index and public open space proximity all indicated greater liveability. Therefore, the negative beta coefficients for these variables revealed decreased liveability in SA2s with higher disability prevalence. By contrast, for distance to a Disability Employment Service, a negative coefficient indicated closer average distance (increased liveability) in SA2s with higher disability prevalence.

No associations were found for the proportion of healthier food options or the average distance to the closest library or CentreLink office (Australia’s social security provider) (Table 2).

Interactions were observed between built environment characteristics and city type for nine built environment variables: distances to physical activity and recreation destination, adult mental health service, dentist, hospital, general practitioner and pharmacy, and walkability index, number of healthier food options and public transport availability (data not shown). Stratified analyses revealed associations in opposite directions for regional cities and major cities (Table 3). In major cities, the direction of association for each of these variables indicated decreased liveability in areas with higher disability prevalence (i.e., greater average distance to specified social infrastructure services, lower walkability, fewer healthier food options and lower public transport availability). In regional cities, by contrast, the direction of association for each of these variables indicated increased liveability in areas with higher disability prevalence.

## 4. Discussion

In this study, we set out to explore associations between disability prevalence in people aged 15–64 years and built environment characteristics at the local (SA2) level in Australia’s 21 largest cities using an ecological analysis approach.

We found that disability prevalence was higher in areas with lower local availability of several social infrastructure services (physical activity and recreation destination, family counselling, adult mental health, psychology, generalist counselling, dentist, activity center, hospital, general practitioner and pharmacy), public open space, healthier food options, and public transport, and with lower walkability. These findings are broadly consistent with the hypothesis that disability prevalence tends to be higher in less liveable and health-promoting neighborhoods.

The only finding that was contrary to this general trend was access to a disability-specific service. The average distance to the closest Disability Employment Service was shorter in areas with higher disability prevalence. Although the association was weak (beta = −0.09), this suggests that there is greater local access to employment support for people with disability who are eligible for this government program in areas with higher disability prevalence. Thus, it appears that Disability Employment Services tend to be located in areas where the need is greater, although we cannot say from our data whether this association reflects effective program targeting or choices made by people with disability to live near disability employment services. In contrast to our findings, a US study found that areas with high numbers of unemployed people with disability were geographically underserved by disability employment support services [61].

There was no association between disability prevalence and variables measuring average distances to the closest library and CentreLink office, or between disability prevalence and healthier food options as a percentage of healthier and fast-food options combined, so no evidence that people with disability tended to live in more or less liveable areas with respect to these amenities. Arguably, it would be desirable to achieve higher local availability of health-promoting neighborhood amenities and infrastructure in areas with higher disability prevalence, as a means of reducing disability-related disadvantage [62].

For nine of the eighteen built environment indicators, the direction of association with disability prevalence differed by city type. Within major cities, disability prevalence was higher in areas with lower local availability of physical activity and recreation destinations, adult mental health services, dentists, hospitals, general practitioners, pharmacies, healthier food options and public transport, and with a lower walkability index. These patterns of lower liveability in areas of higher disability prevalence were not observed within regional cities. As our analysis did not compare liveability between major and regional cities, we cannot comment on whether, in general, there was better local availability of health-promoting neighborhood amenities in major cities or in regional cities. However, the disability-related disadvantage in relation to neighborhood liveability observed within major cities was not apparent within regional cities.

We speculate that the differences in patterns of housing costs in relation to household incomes could explain this finding. Compared with major cities, in regional cities there may be less of a differential in housing costs between areas with different levels of access to neighborhood amenities. If this is so, people with disability may be more likely to be excluded from more liveable areas within major cities than within regional cities. It is notable that disability prevalence was, on average, higher in regional cities (3.5%) than in major cities (2.9%) (Table 1). Existing research indicates that housing can adversely impact health and wellbeing in multiple ways including via associated neighborhood characteristics. Moreover, socioeconomically disadvantaged population groups such as people with disability are particularly vulnerable to such adverse impacts. Housing affordability has been found to be a key factor in driving selective migration of already disadvantaged Australians to less advantaged locations [63,64]. Further research should investigate the role of housing costs and affordability in concentrating the population with disability in less liveable areas within major cities, to inform effective policy responses.

This ecological analysis, utilizing data from the Australian Census and geospatial data on built environment characteristics, has enabled us to identify and describe spatial associations between disability prevalence and aspects of local area liveability. This represents an important contribution to understanding the nature of disability-related disadvantage in Australia. The specific spatial associations we have identified can inform the development of causal models of determinants of health and health inequalities, and indicate possible levers for intervention at the environmental rather than the individual level. From a policy perspective, our findings suggest there may be value in intervening on local built environment characteristics in order to reduce disability-related inequalities in the social determinants of health [65]. Improving access to health-enhancing neighborhood infrastructures may have a particular benefit for people with disability, who are more likely than other groups to experience geographic mobility and cost restrictions and thus to be more reliant on ‘opportunity structures’ available locally [30,66]. For example, for people with disability, increasing neighborhood walkability and local availability of public transport could help facilitate greater social and economic participation. Similarly, increasing local access to public open space, physical activity and recreation destinations, and healthier food options could make it easier to get enough exercise and to eat healthily [37,44]. We suggest that a ‘proportionate universalism’ approach is needed with a scaling of investment in social infrastructure according to disability prevalence [67,68]. Accessibility considerations for people with disability should be an integral aspect of interventions designed to improve local area liveability, promote full participation of people with disability and reduce inequalities [69,70,71].

A number of limitations should be mentioned. First, the regression models were not adjusted for any confounding factors that may explain the observed associations at SA2 level; for instance, population density. However, this paper is mainly descriptive and therefore adjustment for confounding was not considered necessary. Second, a selected list of built environment characteristics was utilized. These measures do not capture all of the aspects of the local built environment that potentially affect health and wellbeing outcomes for the people who live in those areas, and do not account for variation in the quality of environmental amenities (e.g., the quality of services or safety of public open space areas) [72]. Neither do they account for variation in the accessibility of neighborhood services and amenities, including information, communication and physical accessibility facilitators or barriers for people with disability. For example, our indicators of walkability and public transport did not take into account the needs of wheelchair users, and indicators of social infrastructure services did not take into account whether those services were accessible to people with disability. Third, we looked only at associations between built environment characteristics and disability prevalence among people aged 15–64 years, the ‘working age’ population; different patterns of association may be found for disability prevalence among people aged under 15 or over 65 years and thus our findings may not hold for these age groups. Finally, the built environment indicator data were available only for Australia’s 21 largest cities—different patterns of association between disability prevalence and built environment characteristics might be found in rural and remote areas not covered by this study, which represent 20% of Australia’s population aged 15–64 years and include some areas with comparatively higher disability prevalence [73].

Although not a study limitation, it is important to recognize that the Australian Census measure of disability used in this study was designed to be comparable to the ‘profound or severe core activity limitation’ measure available from the Survey of Disability, Ageing and Carers (SDAC), a large survey designed to measure the entire spectrum of disability and the recommended source of disability prevalence data in Australia. Based on data from the 2018 SDAC, the estimated prevalence of disability among those aged 15–64 years was 13% [74], whereas the mean disability prevalence for this age group derived from the Australian Census measure of disability and used in the current study was 3%. Different patterns of association between disability prevalence and built environment characteristics might be observed if it was possible to use the broader SDAC measure of disability.

## 5. Conclusions

The inequitable geographic distribution of health-promoting neighborhood characteristics, as shown by the findings of this study, may be a contributing cause of poorer health and wellbeing outcomes experienced by people with disability. We plan to conduct future research using multilevel modelling techniques to examine the effects of specific built environment characteristics on individual-level health and wellbeing outcomes for people with disability. The selection of outcome areas for this research will be guided by the Disability and Wellbeing Monitoring Framework, which has been developed in consultation with people with lived experience of disability to measure and track inequalities between people with and without disability in relation to exposure to social determinants of health and wellbeing [75]. The framework provides a useful structure within which to consider disability-related disadvantage associated with area-level factors including built environment characteristics and the socio-spatial patterning of social determinants of health and wellbeing.

Policies aiming to redress inequalities should be informed by the spatial distribution of built environment characteristics that are expected to impact social, economic and health outcomes for people with disability [72]. This study provides an important foundation from which to investigate how geographically targeted interventions focusing on built environment characteristics could be used to disrupt the mechanisms by which disability-related disadvantage and consequent inequities are created [65].

## Figures and Tables

**Table 1:** Descriptive statistics for exposure (built environment) and outcome (disability prevalence) variables.

Variables	Range	Median	Mean	Standard Deviation
***Outcome variable*^1^**				
Disability prevalence (15–64 years)—all SA2s (Statistical Area Level 2s) (%)	0–11.9	2.7	3.0	1.5
Major city SA2s (%)	11.9	2.6	2.9	1.5
Regional city SA2s (%)	9.8	3.2	3.5	1.6
***Exposure variables*^2^**				
Number of healthier food options (count)	0–60	5	7	7
Healthier food proportion (%)	0–100	41	43	15
Physical activity and recreation (meters)	265–78,546	4934	2828	6442
Library (meters)	576–22,912	2490	3200	2503
Centrelink (meters)	556–49,384	4361	5364	4127
Disability employment service (meters)	399–26,996	2817	3677	2993
General practitioner (meters)	202–9165	1026	1241	880
Pharmacy (meters)	240–9863	1122	1373	979
Generalist counselling (meters)	289–61,553	2249	3339	3916
Dentist (meters)	241–22,975	1240	1753	1861
Adult mental health (meters)	600–78,935	6419	8891	8602
Family counselling (meters)	475–79,383	4374	6874	8162
Psychology (meters)	242–55,769	1530	2318	3145
Hospital (meters)	294–39,903	3627	5100	4730
Activity center (meters)	236–61,127	1770	2522	3029
Walkability (index)	−7.2–11	0.2	0.3	2.1
Public open space (% dwellings)	0–100	78	74	19
Public transport availability (% dwellings)	0–100	52	52	20

^1^—Source: Census TableBuilder [56]; ^2^—Source: Healthy Liveable Cities group, Centre for Urban Research, RMIT University (https://cur.org.au/research-programs/healthy-liveable-cities-group/).

**Table 2:** Results from the linear regression for the association between built environment indicators (z-standardized variables) and SA2 disability prevalence for people aged 15–64 years.

Variable ^1^	Beta Coefficients	CI low	CI high
Physical activity and recreation	0.29	0.22	0.37
Family counselling	0.28	0.20	0.35
Psychology	0.27	0.20	0.35
Adult mental health	0.24	0.17	0.32
Dentist	0.23	0.15	0.30
Generalist counselling	0.20	0.13	0.28
Activity center	0.18	0.10	0.25
Hospital	0.10	0.03	0.18
General practitioner	0.10	0.02	0.17
Pharmacy	0.10	0.02	0.17
Library	0.06	−0.02	0.14
Healthier food proportion	−0.03	−0.10	0.05
Centrelink	−0.04	−0.12	0.03
Disability Employment Service	−0.09	−0.16	−0.01
Public open space proximity	−0.22	−0.29	−0.14
Walkability	−0.31	−0.39	−0.24
Number of healthier food options	−0.35	−0.42	−0.27
Public transport availability	−0.36	−0.43	−0.29

^1^—Variables are listed in order of beta coefficients.

**Table 3:** Associations between built environment indicators (z-standardized variables) and SA2 disability prevalence for people aged 15–64 years, stratified by city type.

Variable ^1^	Major Cities Beta Coef. (95% CI)		Regional Cities Beta Coef. (95% CI)	
Physical activity and recreation	0.33	(0.26, 0.41)	−0.20	(−0.48, 0.07)
Adult mental health	0.27	(0.20, 0.35)	−0.09	(−0.43, 0.25)
Dentist	0.29	(0.20, 0.37)	−0.07	(−0.24, 0.09)
Hospital	0.15	(0.06, 0.23)	−0.18	(−0.36, 0.00)
General practitioner	0.14	(0.05, 0.23)	−0.10	(−0.24, 0.05)
Pharmacy	0.12	(0.03, 0.20)	−0.08	(−0.24, 0.08)
Walkability	−0.35	(−0.43, −0.28)	0.14	(−0.16, 0.44)
Number of healthier food options	−0.35	(−0.42, −0.27)	0.50	(−0.03, 1.04)
Public transport availability	−0.36	(−0.44, −0.29)	0.38	(−0.06, 0.82)

^1^—Variables are listed in order of beta coefficients for overall associations in Table 2.

## Supplementary Materials

The following is available online at https://www.mdpi.com/1660-4601/17/21/7844/s1, Supplementary file: Correlations between selected built environment indicators.

## Appendix A

**Table A1:** Built environment indicators included in the analysis.

Variable	Definition
Number of healthier food options (count)	Count of ‘healthier’ food options (supermarkets or fruit and vegetable grocers) within 3200 meters of dwelling via pedestrian road network
Healthier food proportion (%)	Healthy food choices percentage—‘healthier’ food options (supermarkets or fruit and vegetable grocers) as percentage of ‘healthier’ and fast-food options combined, within 3200 meters of a dwelling via a pedestrian road network
Physical activity and recreation destination	Average distance to closest physical activity and recreation destination (meters)
Distance to library	Average distance to closest library (meters)
Distance to CentreLink	Average distance to closest CentreLink office (meters). CentreLink is Australia’s national social security provider.
Distance to Disability Employment Service	Average distance to closest Disability Employment Service provider (meters). Disability Employment Services (DES) are an Australian government program that provides assistance with preparing for, finding and keeping employment for people with disability, injuries or health conditions.
Distance to general practitioner	Average distance to closest general practitioner (meters)
Distance to pharmacy	Average distance to closest pharmacy (meters)
Distance to generalist counselling	Average distance to closest generalist counselling service (meters)
Distance to dentist	Average distance to closest dentist (meters)
Distance to adult mental health	Average distance to closest adult mental health services (meters)
Distance to family counselling	Average distance to closest family counselling and/or family therapy service (meters)
Distance to psychology	Average distance to closest psychology service (meters)
Distance to hospital	Average distance to closest hospital (meters)
Distance to activity center	Average distance to closest activity center (meters)
Walkability	Average walkability index for local 1600 meters walkable neighborhoods, relative to study region. The index is calculated as the sum of standardized scores of street connectivity, dwelling density and a daily living score reflecting access to three kinds of basic amenities (a public transport stop, a supermarket and a convenience location). A higher walkability index means better walkability.
Public open space proximity	Percentage of dwellings within 400 meters of public open space
Public transport availability	Percentage of dwellings within 400 meters of public transport stop with a frequent weekday service (at least once every 30 min, 7am–7pm)
