# Supplementary material for: Area-Level Associations between Built Environment Characteristics and Disability Prevalence in Australia: An Ecological Analysis
Source: Int J Environ Res Public Health. 2020 Oct 26;17(21):7844. doi: 10.3390/ijerph17217844 (PMC7662552; doi:10.3390/ijerph17217844)
Supplement: Supplementary file 1 [file ijerph-17-07844-s001.pdf]

**Supplementary file: Correlations between selected built environment indicators**

**Table S1.** Correlation coefficients for food environment indicators.

| | food_02 | food_04 | food_07 | food_06 | food_21 |
|---|---|---|---|---|---|
| **food_04** | 0.6787 | 1 | | | |
| **food_07** | 0.0627 | 0.0319 | 1 | | |
| **food_06** | −0.0338 | −0.1124 | 0.6258 | 1 | |
| **food_21** | −0.3125 | −0.4314 | −0.1335 | 0.0874 | 1 |
| **food_23** | 0.4392 | 0.6115 | 0.0612 | −0.0629 | −0.5261 |

Key to variables:

- food_02: Count of fruit and vegetable grocers within 3200m
- food_04: Count of 'healthier' food options (supermarkets or fruit and vegetable grocers) within 3200m (renamed ‘Number of healthier food options’)
- food_07: Healthy food choices ratio (ratio of healthier to fast-food options within 3200m)
- food_06: Healthy food choices percentage (percentage of 'healthier' relative to healthier and fast-food options combined within 3200m) (renamed ‘Healthier food proportion’)
- food_21: Percentage of dwellings with no availability of healthy or unhealthy food within 3200m
- food_23: Percentage of dwellings within 1000m walking distance of a supermarket

**Table S2.** Correlation coefficients for walkability indicators.

| | walk_20 | walk_19 | walk_18 | walk_nat |
|---|---|---|---|---|
| walk_19 | 0.4874 | 1 | | |
| **walk_18** | 0.4893 | 0.5992 | 1 | |
| **walk_nat** | 0.7403 | 0.8533 | 0.8751 | 1 |
| **walk_city** | 0.7262 | 0.8071 | 0.7965 | 0.9393 |

Key to variables:

- walk_20_hard: Average daily living destination access score for local 1600 metres walkable neighbourhoods
- walk_19: Average street connectivity per square kilometre for local 1600 metres walkable neighbourhoods within area
- walk_18: Average dwelling density per hectare for local 1600 metres walkable neighbourhoods within area
- walk_nat: Average walkability index for local 1600 metres walkable neighbourhoods, relative to 21 cities
- walk_city: Average walkability index for local 1600 metres walkable neighbourhoods, relative to study region (renamed ‘Walkability’)

**Table S3.** Correlation coefficients for public open space indicators.

| | os1_hard |
|---|---|
| **os2_hard** | 0.74 |

Key to variables:

- os1_hard: Percentage of dwellings within 400m of public open space (renamed ‘Public open space proximity’)
- os2_hard: Percentage of dwellings within 400m of public open space > 1.5 Ha area
